# Silicates of Potassium and Aluminium (Kaolin); Comparative Foliar Mitigation Treatments and Biochemical Insight on Grape Berry Quality in *Vitis vinifera* L. (cv. Touriga National and Touriga Franca)

**DOI:** 10.3390/biology9030058

**Published:** 2020-03-20

**Authors:** Rupesh Kumar Singh, Jessica Afonso, Marta Nogueira, Ana A. Oliveira, Fernanda Cosme, Virgílio Falco

**Affiliations:** 1 Centro de Química de Vila Real (CQ-VR), Universidade de Trás-os-Montes e Alto Douro (UTAD), 5000-801 Vila Real, Portugal; jessica.r_afonso@hotmail.com (J.A.); martaandreianogueira@hotmail.com (M.N.); fcosme@utad.pt (F.C.); 2 Departamento de Agronomia, Universidade de Trás-os-Montes e Alto Douro (UTAD), Quinta de Prados, 5000-801 Vila Real, Portugal; anaolive@utad.pt; 3 Centro de Investigação e Tecnologias Agroambientais e Biológicas (CITAB), Universidade de Trás-os-Montes e Alto Douro (UTAD), Quinta de Prados, 5000-801 Vila Real, Portugal

**Keywords:** climate change, mitigation treatments, *Vitis vinifera* L., polyphenols, tannins, anthocyanins

## Abstract

Grapevine physiology is influenced by several environmental factors, such as temperature, precipitation, potential evapotranspiration, and sunshine hours. Due to climatic changes, effects in grapevine physiology and consequently on the grape berry composition and quality have been observed. This work aims to make a comparative study of the effect of foliar mitigation treatment with kaolin (5%) and potassium silicates (0.1% and 0.05%) on the grape berry quality; namely on berry weight, pH, probable alcohol, total phenolics, tannins, total anthocyanins, monomeric anthocyanins, calcium, potassium, and magnesium composition from Portuguese grapevines (*Vitis vinifera* L. cv. Touriga Nacional and Touriga Franca). The results suggested that the phenolic composition and anthocyanin content differs between treatments while other parameters showed distinct behavior among the different applications. Qualitative parameters observed in the present study suggested non-significant changes upon both the applications.

## 1. Introduction

The Douro Demarcated Region is characterized by a typical Mediterranean climate. Climatic changes influence the physiology and phenology of the grapevine, as well as the composition of the grapes. Grapes are mostly affected by solar radiation, temperature, wind, precipitation, humidity, and characteristics of the soil. Mitigation strategies have been evolved to combat extreme climatic conditions to reduce the negative effect on subsequent crop. Particle film is an advanced method of mitigation which incorporates the drizzling of inert clay materials to laminate fruits and leaves surface in the form of a thin film to cope with various climatic conditions [1].

Exogenous foliar application of potassium silicates (K_2_SiO_3_) has been used for disease control in various crops, [2,3,4,5,6,7,8,9] including grapes [10]. These treatments do not improve plant growth or yield; although some differences might have been observed due to indirect effects resulting from the lower infestation of disease causing agents, or due to the presence of some agents within silicates material spray formulations [11].

Aluminium silicate (commonly known as kaolin-Al_2_Si_2_O_5_(OH)_4_)—a radiation-reflecting inert clay material—has been used in particle film technology for pest control for decades [12,13]. Moreover, it has been used in controlling pierce’s disease in viticulture [14], interception of sun burn in fruits [15,16], and to control photon flux density in tree species [17,18]. Its use has been demonstrated as an effective short-term climate change mitigation strategy in Mediterranean vineyards, e.g., by reflecting infrared radiation [1,16], reducing heat stress [19,20,21], and by improving qualitative characteristics [20,22,23,24] in grapevine. Kaolin application has been recently recommended for sustainable viticulture for winegrowers to meet various challenges of changing climatic conditions, especially in Mediterranean-like climates [25].

Foliar exogenous application with silicate materials have been used since the 1990s; however, efficiency and safety issues have been largely overlooked [26]. Therefore, this work aimed to make a comparative study of the efficiency of different foliar mitigation treatment, namely with kaolin and potassium silicate on the grape berries (*Vitis vinifera* L. cv. Touriga Nacional and Touriga Franca) skins and seeds phenolic composition. Field experiments were conducted in Vila Nova de Foz Côa, Douro Demarcated Region in the northern Portugal. Observations of this study may help to understand the silicates-mediated mitigation strategy better, especially for Mediterranean-like climates.

## 2. Materials and Methods

### 2.1. Plant Material, Treatment and Sample Collection

*Vitis vinifera* L. cv. Touriga National and Touriga Franca were the grape varieties studied in the vineyard-Quinta do Orgal situated in Castelo Melhor (longitude 41°3′51.768′ N, latitude 7°5’26.491′ W, altitude 127 to 330 m), Vila Nova de Foz Côa, Douro Superior from the Douro Demarcated Region, northern Portugal in the year 2016 (July to September month). Three phenotypically similar lines were marked (consisting of 12 vines each) for different treatments, i.e., kaolin (5.0%), potassium silicate (0.1%), potassium silicate (0.05%), and control. The vineyard soil has morainic, grass covered, loamy sand with 15% gravel, and is trained according to the Guyot system. The weather conditions during the year have been presented in the Supplementary Table S1.

**Figure 1:**
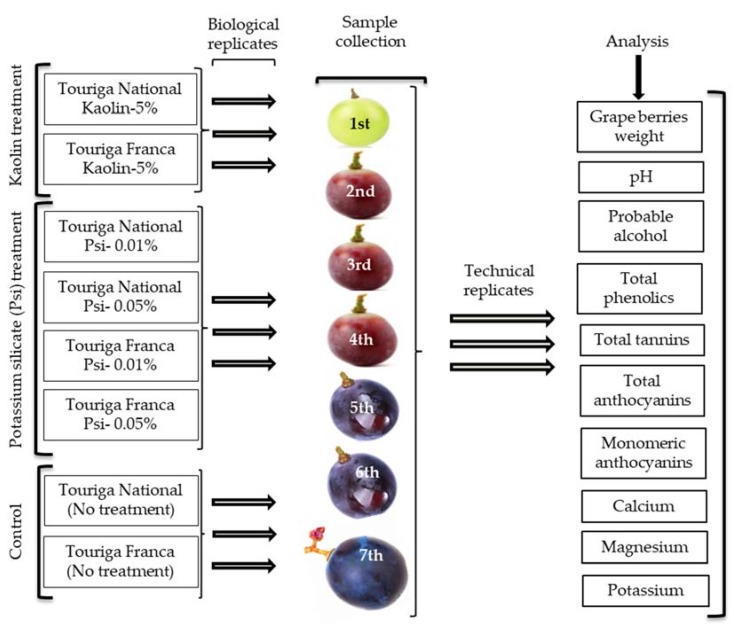
Diagrammatic representation of experimental setup in the field.

Kaolin and potassium silicate solutions were prepared and applied (leaves and berries) in both grape varieties while three similar lines were maintained as control. All applications were performed manually by using sprayer. All the applications were performed at the beginning of veraison (as soon as the berries start coloring). Leaves and berries were ensured to be covered completely as white particle film of a kaolin solution. Potassium silicate application was performed similarly on leaves and berries. Berries were collected randomly in duplicates during seven different time intervals since veraison until final maturation. Berries were dissected into berry skins, seeds, and pulp from all the treated grapevines, as well as the control grapevines. Berry skins and seeds were frozen and freeze-dried for extracts preparations, while pulp was crushed into fine juice and stored in −20° C for further analysis. Complete experimental set up in the field area has been described diagrammatically in Figure 1.

### 2.2. Grape Berry Weight, pH, and Probable Alcohol

A total of 200 berries was collected randomly from all treated grapevines as well as from control plants. Weight of individual berry and mean weight was recorded by analytical balance (KERN, GmBH, Germany). The pulp was crushed into fine juice by using a homogenizer (T25 digital ULTRA-TURRAX^®^, IKA, GmBH, Germany) and pH was recorded by digital pH meter (TitroLine^®^7000, Hertfordshire, UK). The probable alcohol was determined by reading the refractive index of the homogenized pulp by using digital refractometer with automatic temperature compensation (Hanna HI 96813, Bedfordshire, UK) and presented as % *v*/*v*.

### 2.3. Calcium, Magnesium, and Potassium

The concentration of potassium, magnesium, and calcium was determined in berry pulps by atomic absorption spectrometry with flame atomizer (FAAS, PerkinElmer 4110 ZL, USA). Grape pulps were centrifuged in a 1.5 mL eppendorf tube at 11,000 rpm for 25 mins at 25 °C, and the resulting supernatant was collected. Calcium evaluation was done by the addition of 200 μL of the sample, 5.8 mL of ultra-pure water, and 0.45 mL of strontium chloride (5% *v*/*v*). Potassium and magnesium were done by mixing sample (100 μL), 20 mL of ultra-pure water, and 0.4 mL of cesium chloride (5%). Calibration standards were run first followed by all the extracts, absorbance was detected and concentration was calculated using the least square method in all samples [27].

### 2.4. Extract Preparation

Extracts were prepared from 100 mg of grounded tissue from all the samples in triplicates. Grounded tissue was mixed with 3.0 mL of 50% aqueous ethanol and allowed to mix in the agitator for 1 h at room temperature. Samples were then centrifuged at 10,000 rpm at 4 °C for 20 min and supernatant was taken out in a separate tube. Extracts were stored in −20 °C until further analyses.

#### 2.4.1. Total Phenolics, Tannins, and Anthocyanin Content

Methods to analyse total phenolics and total anthocyanins (grape berry skins) was adopted from previously established protocols with slight modifications [27,28]. In brief, 200 µL of aqueous ethanolic extract was mixed with 3.8 mL of 1.0 M aqueous hydrochloric acid by gentle shaking. The mixtures were allowed to incubate at 28 °C for 2 h and absorbance was recorded at 520 nm and 280 nm by spectrophotometer (Evolution 201, Thermoscientific, USA). Values were calculated and expressed as mg of malvidin-3-*O*-glucoside equivalent per mg dry weight for anthocyanin content, and mg of epicatechin equivalent per mg dry weight for total phenolic content. All experiments were done in triplicates to minimize the standard error.

Aqueous ethanolic extract (200 µL) from skins and seeds were mixed with 0.04% methyl cellulose solution and incubated at room temperature for 3 min. A saturated solution of ammonium sulphate (400 µL) was added and volume was made up to 2 mL with deionised water. Separate corresponding control was prepared by replacing methyl cellulose solution with water. Mixtures were centrifuged at 4000 rpm for 5 min, the supernatant was taken out in separate tubes, absorbance was recorded at 280 nm, and total tannin content was calculated and expressed as mg epicatechin equivalent per mg dry weight using the calibration curve. The method described above was adopted from Mercurio et al. [29] and Sarneckins et al. [30] with slight modifications.

#### 2.4.2. Monomeric Anthocyanins Quantification by HPLC

HPLC analysis was performed by using Gilson chromatograph equipped with a Thermo Finnigan Surveyor PDA plus detector. Kinetex C18 columns were used (150 mm long, 4.6 mm in diameter, 5 μm particle size). The details of the two mobile phases, A and B are as follows: mobile phase A consisted of ultra-pure water, formic acid and acetonitrile in the proportions 87:10:3 (*v*/*v*/*v*) respectively; and the mobile phase B consisting of ultra-pure water, formic acid and acetonitrile in the proportions 40:10:50 (*v*/*v*/*v*), respectively. Before being used for analysis, the samples were filtered through a 0.2 µm membrane filter. Detection was performed at 518 nm with an injection volume of 20 μL, a flow rate of 0.8 mL/min, and a temperature of 40 °C for a running time of 45 min. Monomeric anthocyanins were quantified according to the peak areas obtained in the chromatograms. Calibration curves were obtained with cyanidin-3-*O*-glucoside, malvidin-3-*O*-glucoside, peonidin-3-*O*-glucoside, and pelargonidin-3-*O*-glucoside. Using the coefficient of molar absorptivity (ε) and by extrapolation, it was possible to obtain the slopes for delphinidin-3-*O*-glucoside (ε = 23,700 L/mol/cm), petunidin-3-*O*-glucoside (ε = 18,900 L/mol/cm), and malvidin-3-*O*-coumaroylglucoside (ε = 20,200 L/mol/cm) to perform the quantification. The results of delphinidin-3-*O*-acetylglucoside, petunidin-3-*O*-acetylglucoside, peonidin-3-*O*-acetylglucoside, cyanidin-3-*O*-acetylglucoside, and cyanidin-3-*O*-coumarylglucoside were expressed as respective glucoside equivalents.

### 2.5. Statistical Analysis

Two replicates were taken from the field were considered as “biological”, and the three from the lab as “technical” and expressed as mean ± standard deviation. Statistically significant differences between means were determined by analysis of variance (ANOVA, one-way) followed by Tukey’s honestly significant difference (HSD, 5% level) post-hoc test. All analyses were performed using Statistica 7 software (StatSoft, Tulsa, OK, USA).

## 3. Results

The present investigation was aimed to study the comparative mitigation effect of kaolin and potassium silicate on two different Portuguese red grapevines (variety Touriga National and Touriga Franca) in Douro Demarcated Region. The grape berries were collected from each treatment as well as control and analyzed concerning different parameters. The collection of the grape berries began on July 26, 2016, followed by days 2, 9, 17, and 23 August and finally 7 and 20 September (final harvesting).

### 3.1. Weight of the Berries

Grape berries from each treatment were randomly collected in duplicate on different days after treatment; as well as from control grapevine lines. A total of 100 berries were collected randomly in duplicates, and the weight of each berry was determined, and the mean weight was calculated and presented in Table 1. Mean values indicated an increasing pattern in berry weight during maturation for both grape varieties. Observation suggested that Touriga National variety presents the lower weight of grape berries than Touriga Franca; although, no significant difference was observed in each grape variety in all treatments at final maturation in comparison to the control.

### 3.2. pH Values

The grape berries were separated into skin, seeds, and pulp. The pulp was crushed into fine juice and the pH was determined and presented in the Table 2. Gradual increase along maturation was observed but potassium silicate treatment showed no difference in pH in the Touriga National variety, while a slight decrease was recorded upon kaolin treatment. Touriga Franca variety showed a slight increase in pH values upon potassium silicate treatment, whereas a slightly lower pH upon kaolin treatment was recorded. However, no significant difference was observed in each grape variety in all treatments at final maturation in comparison to control.

### 3.3. Probable Alcohol

Probable alcohol was measured in pulps of all samples and it showed a gradual increase during maturation in both grape varieties (Table 3). Treated grapevines showed a slight decrease in the probable alcohol in Touriga National in comparison to the control while Touriga Franca showed a slight increase in all treatments, especially in the grape berries with potassium silicate treatment in both concentrations. Although no significant difference was observed in each grape variety in all treatments at final maturation in comparison to control.

### 3.4. Total Phenolics

Phenolic compounds are very important for grape berry quality due to their nutraceutical and health benefit properties. An increase in the total phenolics were recorded in the Touriga National grape berries throughout the maturation in control as well as in all the treated grapevines (Table 4). Moreover, there was no significant difference observed in Touriga National grape berries upon any treatment in comparison to control at final maturation data. In contrast, Touriga Franca grape berries showed a differential pattern in phenolic compounds accumulation during the maturation process. Although kaolin treated grapevines of Touriga Franca reflected a small increase in phenolic compounds but potassium silicate showed a slight decrease in both the concentrations in comparison to control (Table 4). However, there was no significant difference observed in Touriga Franca grape berries upon any treatment in comparison to control at final maturation data. Moreover, Touriga Franca berries contained higher phenolics in comparison to the Touriga National in control as well as in all the treatments. Highest total phenolic content in the Touriga Nacional corresponds to the berries subjected to treatment with 0.1% potassium silicate (50.9 mg/g dry wt) was comparable to the control sample (46.1 mg/g dry wt), and to the kaolin-treated berries (67.2 mg/g dry wt) in Touriga Franca. Although these differences were not significantly different when compared to the untreated sample in both grape varieties at final maturation in comparison to control.

### 3.5. Total Tannins in Berry Skins and Seeds

Astringency is a peculiar characteristic of grape berries and its derived products, which depends on the tannin content (Table 5). The highest concentration of the total tannins for both grape varieties was recorded on August 9 and started decreasing until the final maturation. Touriga Nacional grape variety treated with 0.05% potassium silicate had a higher total tannin content (33.62 mg/g dry wt) compared to the control (32.36 mg/g dry wt), on the date of final harvesting. While in Touriga Franca grape variety, the highest concentration was recorded in kaolin-treated samples (49.77 mg/g dry wt). However, the tannin content at the time of final harvesting was not significantly different among the different treatments in either Touriga Nacional or Touriga Franca grape variety.

Total tannin content was also analyzed in grape seeds as well upon all the treatments in comparison to control in both the grape varieties (Table 6). The highest concentration was found in grape seed samples treated with 0.05% potassium silicate for the Touriga Nacional (87.94 mg/g dry wt) and the Touriga Franca as well (127.68 mg/g dry wt). However, the tannin content in grape seeds at the time of final harvesting was not significantly different among the different treatments in either Touriga Nacional or Touriga Franca grape variety.

### 3.6. Total Anthocyanins in Berry Skins and Monomeric Anthocyanins

Total anthocyanin content was analyzed in the grape berry skins of both grape varieties under all the treatments and compared to the control grapevines (Table 7). Touriga Nacional showed a tendency of a significant increase in the total anthocyanins over the maturation time, and at final harvesting time a higher concentration of anthocyanin was recorded. Total anthocyanins in grapevines treated with 0.05% potassium silicate (9.42 mg/g dry wt of berry skin) compared to control sample (8.07 mg/g dry wt of berry skin) demonstrated the highest value of total anthocyanins. However, data presented in the Table 7 for Touriga Nacional grape variety demonstrated that no significant difference was recorded between the different treatments and control at the time of final harvesting.

On the contrary, Touriga Franca showed a gradual and significant increase in total anthocyanins as on 23 August (at veraison cessation) and then a slight decrease in the concentration of these compounds. The highest value of total anthocyanins was recorded in kaolin treated grapevines (11.37 mg/g dry wt). Grapevines treated with 0.1% potassium silicate revealed the lowest value for these compounds (8.59 mg/g dry wt).

However, also in the total anthocyanins, there were no significant differences among all the trials at the time of final maturation in both varieties studied.

Various monomeric anthocyanins (a total of 14) in different forms (glycosylated, acetylated, and coumarylated) were identified and quantified in berry skins of both the varieties by using HPLC (Table 8). The identification and quantification of monomeric anthocyanins were performed only in samples collected at the time of the final harvest. Berry skins of the Touriga Nacional and the Touriga Franca grapevines subjected to different types of treatment showed the highest concentration of glycosylated anthocyanins. Malvidin-3-glucoside was observed as the predominant anthocyanin. In general, for both varieties, glycosylated anthocyanins marked an increase in their concentration in the samples where the leaf treatments were applied. In acetylated anthocyanins, delphinidin-3-*O*-acetylglucoside was not detected in any of the studied grape varieties, either for the treated or control samples. In general, there was also an increase in the concentration of acetylated anthocyanin in the treated samples compared to the control samples, except for the Touriga Franca malvidin-3-*O*-acetylglucoside, which showed a higher concentration in the control samples.

Delphinidin-3-*O*-coumaroylglucoside, a coumarylated anthocyanins, was detected only in the samples treated with kaolin in Touriga Nacional grape berries. Cyanidin-3-*O*-coumaroylglucoside was recorded in a higher concentration in treated samples, while the remaining coumarylated monomeric anthocyanins were decreased in their concentration compared to control samples. In Touriga Franca grape berries, there was an increase in its concentration except for malvidin-3-*O*-coumaroylglucoside where there was a decrease in the concentration of the samples subjected to both types of leaf treatment.

### 3.7. Calcium, Potassium, and Magnesium Content

Calcium concentration was analyzed in the grape pulp of both the grape varieties to evaluate the influence of foliar applications (Table 9). Touriga Nacional grape pulp samples recorded a significantly higher concentration of calcium (98.4 mg/g dry wt) in 0.05% potassium silicate treatment, followed by 0.1% potassium silicate treated grapevines (69.0 mg/g dry wt), and was recorded the lowest in the kaolin treatment (65.0 mg/g dry wt) when comparison to the control. The significant difference was observed only for the treatment with 0.1% potassium silicate in comparison to the control at the final maturation data. On contrary, kaolin treated grapevines in Touriga Franca grape variety showed an increase (68.6 mg/g dry wt) in comparison to the control. Moreover, both potassium silicate applications also increased this parameter in the Touriga Franca as well, however, there was no significant difference observed upon any treatment in comparison to control at final maturation data.

Potassium concentration was analyzed in the grape pulp of both grape varieties and presented in the Table 10. Touriga Nacional grape pulp samples recorded the highest concentration of potassium (1113.7 mg/g dry wt) when treated with 0.05% potassium silicate, followed by a decrease in 0.1% potassium silicate treated grapevines (806.5 mg/g dry wt), and it was found the lowest in kaolin treatment (792.7 mg/g dry wt), in comparison to the control. On the other hand, 0.1% potassium silicate treated grapevines in the Touriga Franca variety recorded the highest potassium (1446.1 mg/g dry wt), and 1384.7 mg/g dry wt upon 0.05% potassium silicate treatment in comparison to the control. Moreover, kaolin treatment recorded lower concentration (853.0 mg/g dry wt) in the Touriga Franca variety. However, there was no significant difference observed in both the grape varieties upon any treatment in comparison to the control at the final maturation data.

The concentration of magnesium was also analyzed in the grape pulp of both grape varieties and presented in the Table 11. Touriga National samples showed an increase in all the three treatments in comparison to the control, whilst its highest concentration was recorded in 0.05% potassium silicate treatment. Similarly, the Touriga Franca variety showed the highest quantity of magnesium in 0.05% potassium silicate treatment followed by 0.1% treated grapevines. Kaolin treated Touriga Franca grapevines did not exhibit a distinct difference in comparison to the control. However, there was no significant difference observed in both grape varieties upon any treatment in comparison to control at final maturation data.

## 4. Discussion

The mean weight observation indicated that the Touriga National grape variety presents a lower weight of grape berries than the Touriga Franca. This differential behavior suggested that the Touriga Franca grape variety has a higher resistance to the conditions of mitigation in the region where it was collected, as it adapts better to warmer and drier climates [31]. Treatments showed lower berry weights in both the grape varieties than control. Different mitigation conditions during the growth of the berries were studied earlier and the water deficit condition was observed to reduce the average mass of the berries [32]. Manual pruning was studied in the Dão wine region in Touriga Nacional grape variety and reported higher berry weight [33]. Influence of the way of conduction on the productivity and quality of grape berries were studied and subsequent changes resulted in a higher berry mass [34]. Several studies observed different berry mass in the different climate zone upon different mitigation strategies and observed an effect of varietal dependency in particular climatic conditions [35,36,37,38]. Kaolin showed an inhibitory effect in grape berry weight in the Cabernet Sauvignon cultivar [39], while another study showed an increase in grape berry weight upon its treatment [21]. Potassium silicate, as well as other silicates, did not influence the berry weight and production [26] in earlier studies. Present investigation suggests that kaolin and potassium silicate has no stimulatory effect on the berry weight and production. Kaolin is a silicate of aluminium, which may increase the surface temperature of berries during strong sunlight and thus increase the transpiration rate as noticed by a 1 °C increase in the tomato temperature upon kaolin spray [15].

Furthermore, the grape pulp juice was prepared from all the treated grapevines as well as from the control and the pH was determined, and a gradual increase along maturation was observed. However, potassium silicate treatment showed no difference in the pH. Although kaolin treatment showed a slight decrease in pH in both grape varieties and supports the recent findings in Cabernet Sauvignon cultivar [39]. Previously, winemaking behavior of Touriga National grape variety was studied in Douro Demarcated Region and a similar pH range was observed as in present study [40]. A recent report demonstrated lower pH values than those recorded in the present study while studying the influence of the way of conduction [34]. The strategic effects of deficit irrigation on the yield and quality of the Touriga Franca variety in the Douro region also showed lower pH values. The present study showed similar observations as reported by Lima [36] in the Douro region and by Sereno [41] in the Tagus region, whereas the other reports noticed a lower range of pH.

Probable alcohol was measured in all the berries and showed a gradual increase during maturation in both the grape varieties, indicating that mitigation measures did not influence this parameter in the Douro region (Table 4). Recent mitigation study demonstrated a lower value of probable alcohol in the Touriga National grapevine in comparison to the present study [34]. While other reports observed a 14.0% [35], 11.40% [38], 12.5% [42], and 9.80% to 14.80% [43] probable alcohol content in the Touriga National.

Shellie and King [24] and Lobos et al. [44] found that the kaolin-treated grapevines had grapes with a higher content of the total phenolic compounds, which is also verified in the results obtained in this study, for both grape varieties with a very low quantitative shift, although these differences are not significant when compared to the untreated sample. In studies by Dinis et al. [20], it was found that samples subjected to kaolin treatment have higher total tannin content. In this study, this is only verified for Touriga Franca, unlike Touriga Nacional, where it is the samples treated with 0.05% potassium silicate that has the highest tannin concentration. However, this difference is not significant compared to control (Table 9). In the remaining samples, and for both grapevines varieties, the highest total tannin content was verified in the samples of the seeds treated with 0.05% potassium silicate. Brillante et al. [39] performed a three year comparative study for kaolin and pinolene application. A higher total flavonoid accumulation was recorded in the first year, which decreased significantly in the subsequent two years. There was a constant increase in the total anthocyanins in all the three years of kaolin application. While the similar study demonstrated a lower berry weight and yield per grapevine during all three years upon kaolin application. The anthocyanin content for samples subjected to kaolin treatment was higher in present study, as for the Touriga Nacional and Touriga Franca grapes, although, as already mentioned, this increase was not significant in relation to the control.

The total phenols, flavonoids and anthocyanins were studied upon kaolin application and a significant increase was reported in the berries at the final maturation [20]. These findings suggest that the lower reactive oxygen species and the increased non enzymatic antioxidants in leaf tissues and berries might be due to the increased phenolic and anthocyanin compounds. A positive correlation was assumed between higher phenolics and antioxidants most likely caused by a stimulatory effect of phenylpropanoids pathway at the molecular level. Higher kaolin doses of up to 60 g/L was applied in other studies with a wetting of 950 L/ha [16,24]. The effects of kaolin application were observed differently and were found mainly influenced by the grapevine water status. For example, well irrigated grapevines showed reduced canopy temperature, higher leaf water potential, and lower gases with slight fruit compositional variations depending on the cultivar. It has previously been reported that kaolin did not change the photosynthetic rate [16,45], moreover; Morandi et al. [46] and Otero et al. [47] did not observe any significant alteration in the rate of transpiration. Findings of the present study were similar with those by Glenn et al. [16], where no significant differences were recorded upon kaolin applications on Merlot and Viognier grape varieties. Other reports noticed that kaolin negates the negative effect of water stress and photoinhibition in a warmer climate indirectly [48]. Finally, no differences were recorded in berry composition upon kaolin treatment, although it may help to combat the negative effects of hot temperature, especially in warmer climatic conditions in grape berries [44]. Some of the individual monomeric anthocyanins did show improvement upon the treatment. Nonetheless, there is no report on the individual anthocyanin analysis earlier on these applications, and the present study investigates these effects for the first time in the best of our knowledge.

Most cations are transported by xylem sap, which is directly related to the amount of water transpired by the plant. After the veraison, due to changes in grape skin and stomatal degeneration, the intensity of perspiration decreases gradually, which presupposes a similar decrease in cation translocation [49], explaining the sharp decrease in calcium concentration on August 2, the date when the veraison began. Donéche and Chardonnet [50] reported that during the ripening there is an accumulation of calcium in the berries. However, this is not verified in the present study, although the effect was more evident in the Touriga Nacional variety. Earlier work in the Serra Gaúcha grape variety found a calcium concentration of 68.4 mg/L in the grape must, which was similar to those obtained in the present study [51].

In this study, an increase in potassium concentration in the last date of harvesting has been recorded. Potassium allows for the displacement of sugars derived from photosynthesis, and as there is an increase in sugars, potassium accumulation in the berry is potentiated [52]. Phloem allows translocation of sugars from photosynthesis. Moreover, potassium is also one of the rare mineral elements carried by sap through this pathway. Thus, during maturation, the increase of potassium concentration in the grapes was directly related to the kinetic accumulation of sugar [49], as verified in the present study and by the work of Rizzon and Miele [51] on grape must. Borges [53] indicated an average value of potassium concentration in the must in the order of 1000 mg/L. The present study recorded similar values for the pulp of Touriga Franca grapes, and lower for Touriga Nacional grapes pulp, while the kaolin application has lower values than the control in both the varieties.

Analysis of the concentration of magnesium content revealed that the foliar applications do not influence the behavior of magnesium transport in the grapevine, with an exception of potassium silicate (0.05%) application, which showed a higher accumulation in both the varieties at the final maturation. Szöke et al. [54] found a slight accumulation of magnesium at the beginning of the veraison similar to the present study. Pacheco et al. [55] and Parejo et al. [56] reported that the magnesium content decreased in berries as in the present study. The average value of the magnesium concentration in the must, as determined by Borges [53], was 150 mg/L, which was much higher than those obtained in the present study in the two varieties, and in the different treatments performed.

## 5. Conclusions

Present results showed that the average weight and the pH of the grape berries are not influenced by the mitigation conditions. In addition, although some improvement has been recorded in treated grapevines; the differences were however not significant when compared with the control. Although, total anthocyanins and monomeric anthocyanins were influenced by the foliar applications. In addition, a differential profile was recorded in calcium, potassium, and magnesium content, which changed with mitigation treatments. The results obtained are of great interest, since it allows a better understanding of these treatments as a measure to protect grapevines from the severe droughts and the high sun exposure in the recent times. Non-significant changes in the quality of the grape berries questions the potential applications of such treatments in the vineyards for qualitative improvements.

## Figures and Tables

**Table 1:** Grape berry weight (g) of Touriga Nacional and Touriga Franca varieties during different times of sampling until maturation.

Variety/Sampling Time	Control	Kaolin 5%	Potassium Silicate (0.1%)	Potassium Silicate (0.05%)
**Touriga National**				
26/07	0.68 ± 0.06 ^aA^	0.65 ± 0.06 ^aA^	0.54 ± 0.01 ^aA^	0.57 ± 0.04 ^aA^
02/08	0.73 ± 0.05 ^aA^	0.63 ± 0.08 ^aA^	0.59 ± 0.02 ^aA^	0.62 ± 0.14 ^abA^
09/08	0.81 ± 0.05 ^ab^	0.70 ± 0.04 ^aA^	0.62 ± 0.09 ^aA^	0.67 ± 0.11 ^abA^
17/08	1.00 ± 0.08 ^bcA^	1.06 ± 0.01 ^bA^	0.96 ± 0.02 ^bA^	0.86 ± 0.12 ^abA^
23/08	0.99 ± 0.02 ^bcA^	1.06 ± 0.03 ^bA^	1.04 ± 0.11 ^bA^	0.92 ± 0.04 ^abA^
07/09	1.25 ± 0.00 ^dA^	1.11 ± 0.15 ^bA^	0.94 ± 0.02 ^bA^	1.10 ± 0.21 ^bA^
20/09	1.19 ± 0.09 ^cdA^	1.18 ± 0.11 ^bA^	1.09 ± 0.13 ^bA^	1.05 ± 0.13 ^abA^
**Touriga Franca**				
26/07	1.01 ± 0.11 ^aA^	0.92 ± 0.04 ^aA^	0.92 ± 0.19 ^aA^	1.04 ± 0.02 ^aA^
02/08	1.101 ± 0.33 ^abA^	1.19 ± 0.01 ^aA^	1.12 ± 0.21 ^abA^	1.05 ± 0.15 ^aA^
09/08	1.51 ± 0.16 ^abcA^	1.20 ± 0.41 ^bA^	1.16 ± 0.23 ^abA^	1.05 ± 0.48 ^aA^
17/08	1.59 ± 0.04 ^abcA^	1.54 ± 0.01 ^abA^	1.60 ± 0.17 ^bA^	1.28 ± 0.080 ^aA^
23/08	1.69 ± 0.02 ^bcA^	1.47 ± 0.17 ^abA^	1.43 ± 0.12 ^abA^	1.32 ± 0.04 ^aA^
07/09	1.58 ± 0.03 ^abcA^	1.52 ± 0.03 ^abA^	1.41 ± 0.03 ^abA^	1.43 ± 0.14 ^aA^
20/09	2.01 ± 0.20 ^cA^	1.59 ± 0.19 ^abA^	1.56 ± 0.04 ^baA^	1.59 ± 0.02 ^aA^

Means (average ± standard deviation) within a column followed by different lower case letters are significantly different (*p* < 0.05). Values with the same capital letter on the same line for each grape variety and sampling data are not significantly different from each other at *p* < 0.05.

**Table 2:** The pH of grape must of Touriga Nacional and Touriga Franca grape variety during different time of sampling until maturation.

Variety/Sampling Time	Control	Kaolin 5%	Potassium Silicate (0.1%)	Potassium Silicate (0.05%)
**Touriga National**				
26/07	2.77 ± 0.02 ^aA^	2.79 ± 0.02 ^aA^	2.78 ± 0.04 ^aA^	2.77 ± 0.04 ^aA^
02/08	2.93 ± 0.09 ^aA^	2.84 ± 0.04 ^abA^	2.83 ± 0.01 ^aA^	2.89 ± 0.04 ^abA^
09/08	3.05 ± 0.00 ^abA^	2.97 ± 0.06 ^abcA^	3.00 ± 0.06 ^aA^	2.96 ± 0.08 ^abA^
17/08	3.46 ± 0.00 ^cA^	3.22 ± 0.04 ^bcA^	3.33 ± 0.03 ^bA^	3.28 ± 0.16 ^bcA^
23/08	3.37 ± 0.16 ^bcA^	3.35 ± 0.11 ^cA^	3.54 ± 0.04 ^bA^	3.48 ± 0.22 ^cA^
07/09	3.91 ± 0.16 ^dA^	3.89 ± 0.20 ^dA^	4.06 ± 0.12 ^cA^	3.95 ± 0.04 ^dA^
20/09	4.19 ± 0.08 ^dA^	4.07 ± 0.11 ^dA^	4.21 ± 0.09 ^cA^	4.26 ± 0.00 ^dA^
**Touriga Franca**				
26/07	2.86 ± 0.07 ^aA^	2.86 ± 0.01 ^aA^	2.91 ± 0.03 ^aA^	2.88 ± 0.07 ^aA^
02/08	2.99 ± 0.02 ^aA^	3.06 ± 0.03 ^abA^	3.07 ± 0.08 ^aA^	3.10 ± 0.03 ^aA^
09/08	3.23 ± 0.00 ^bA^	3.22 ± 0.01 ^abA^	3.25 ± 0.25 ^aA^	3.27 ± 0.18 ^aA^
17/08	3.68 ± 0.14 ^cA^	3.68 ± 0.12 ^bcA^	3.84 ± 0.08 ^bA^	3.81 ± 0.12 ^bA^
23/08	3.60 ± 0.00 ^cA^	3.65 ± 0.21 ^bcA^	4.01 ± 0.06 ^bA^	3.99 ± 0.11 ^bcA^
07/09	4.06 ± 0.01 ^dA^	3.92 ± 0.24 ^cA^	4.21 ± 0.01 ^bA^	4.30 ± 0.13 ^cdA^
20/09	4.34 ± 0.01 ^eA^	4.29 ± 0.25 ^cA^	4.67 ± 0.01 ^cA^	4.71 ± 0.06 ^dA^

Means (average ± standard deviation) within a column followed by different lower case letters are significantly different (*p* < 0.05). Values with the same capital letter on the same line for each grape variety and sampling data are not significantly different from each other at *p* < 0.05.

**Table 3:** Probable alcohol (%*v*/*v*) in grape berries of Touriga Nacional and Touriga Franca grape variety during different time of sampling until maturation.

Variety/Sampling Time	Control	Kaolin 5%	Potassium Silicate (0.1%)	Potassium Silicate (0.05%)
**Touriga National**				
26/07	5.6 ± 0.1 ^aA^	5.4 ± 0.2 ^aA^	5.4 ± 0.1 ^aA^	5.4 ± 0.2 ^aA^
02/08	8.4 ± 0.1 ^bA^	7.8 ± 0.8 ^bA^	8.6 ± 0.6 ^bA^	8.4 ± 1.6 ^abA^
09/08	12.8 ± 1.5 ^cdA^	12.2 ± 1.8 ^dA^	11.0 ± 0.2 ^cA^	11.5 ± 1.7 ^bcA^
17/08	9.6 ± 0.2 ^bcA^	11.2 ± 0.1 ^cA^	10.6 ± 0.1 ^cA^	11.3 ± 0.7 ^bcA^
23/08	10.6 ± 1.4 ^bcdA^	11.7 ± 0.1 ^cA^	11.6 ± 0.1 ^cdA^	11.5 ± 0.4 ^bcA^
07/09	12.8 ± 0.9 ^cdA^	12.8 ± 0.1 ^dA^	13.5 ± 0.1 ^eA^	13.4 ± 0.6 ^cA^
20/09	13.4 ± 0.0 ^dA^	13.0 ± 0.1 ^dA^	12.7 ± 0.5 ^deA^	12.9 ± 0.0 ^cA^
**Touriga Franca**				
26/07	5.6 ± 0.1 ^aA^	5.2 ± 0.9 ^aA^	5.4 ± 0.7 ^aA^	5.5 ± 0.6 ^aA^
02/08	9.3 ± 0.3 ^bA^	6.7 ± 0.3 ^abA^	7.4 ± 2.5 ^abA^	6.5 ± 2.8 ^abA^
09/08	9.1 ± 0.1 ^bA^	8.3 ± 0.8 ^bA^	8.8 ± 2.3 ^bA^	9.0 ± 2.6 ^bA^
17/08	10.2 ± 0.6 ^cA^	10.6 ± 1.2 ^bA^	10.4 ± 0.0 ^cA^	11.3 ± 0.0 ^cA^
23/08	11.0 ± 1.1 ^cA^	11.0 ± 0.7 ^bcA^	12.2 ± 0.8 ^cdA^	11.0 ± 0.6 ^cA^
07/09	11.9 ± 0.6 ^dA^	12.4 ± 0.8 ^cA^	13.0 ± 0.1 ^dA^	13.4 ± 0.1 ^dA^
20/09	12.2 ± 0.1 ^dA^	12.5 ± 0.6 ^cA^	13.0 ± 0.8 ^dA^	13.2 ± 0.3 ^dA^

Means (average ± standard deviation) within a column followed by different lower case letters are significantly different (*p* < 0.05). Values with the same capital letter on the same line for each grape variety and sampling data are not significantly different from each other at *p* < 0.05.

**Table 4:** Total phenolics (mg/g) in grape berries of Touriga Nacional and Touriga Franca grape variety during different time of sampling until maturation.

Variety/Sampling Time	Control	Kaolin 5%	Potassium Silicate (0.1%)	Potassium Silicate (0.05%)
**Touriga National**				
26/07	37.98 ± 0.46 ^aA^	33.62 ± 0.75 ^aA^	35.33 ± 2.53 ^aA^	33.17 ± 2.54 ^aA^
02/08	35.66 ± 2.42 ^aA^	33.68 ± 0.99 ^aA^	36.03 ± 2.26 ^aA^	35.95 ± 1.72 ^aA^
09/08	41.88 ± 4.66 ^aA^	43.37 ± 11.83 ^aA^	42.26 ± 6.57 ^aA^	39.71 ± 4.36 ^aA^
17/08	38.78 ± 2.53 ^aA^	39.47 ± 3.04 ^aA^	43.73 ± 9.21 ^aA^	43.26 ± 13.09 ^aA^
23/08	39.82 ± 9.33 ^aA^	41.19 ± 6.51 ^aA^	47.87 ± 11.05 ^aA^	51.24 ± 6.69 ^aA^
07/09	44.16 ± 3.47 ^aA^	42.42 ± 8.05 ^aA^	44.81 ± 15.83 ^aA^	41.76 ± 5.82 ^aA^
20/09	46.18 ± 8.18 ^aA^	50.96 ± 1.07 ^aA^	52.07 ± 11.28 ^aA^	51.32 ± 4.97 ^aA^
**Touriga Franca**				
26/07	53.53 ± 1.09 ^aA^	49.33 ± 1.74 ^aA^	49.17 ± 8.13 ^aA^	52.21 ± 3.59 ^aA^
02/08	53.48 ± 9.94 ^abA^	52.71 ± 4.65 ^aA^	58.09 ± 4.46 ^aA^	54.07 ± 1.35 ^aA^
09/08	84.81 ± 6.06 ^abA^	67.55 ± 12.80 ^aA^	67.57 ± 2.79 ^aA^	58.78 ± 18.07 ^aA^
17/08	67.46 ± 13.31 ^abA^	74.12 ± 1.90 ^aA^	68.69 ± 5.08 ^aA^	61.25 ± 7.54 ^aA^
23/08	68.29 ± 3.97 ^abA^	70.96 ± 17.97 ^aA^	65.82 ± 16.04 ^aA^	62.51 ± 0.46 ^aA^
07/09	81.62 ± 9.66 ^abA^	74.97 ± 9.29 ^aA^	57.07± 3.96 ^aA^	59.48 ± 10.74 ^aA^
20/09	66.48 ± 3.58 ^bA^	67.22 ± 0.90 ^aA^	55.39 ± 6.79 ^aA^	54.87 ± 11.39 ^aA^

Means (average ± standard deviation) within a column followed by different lower case letters are significantly different (*p* < 0.05). Values with the same capital letter on the same line for each grape variety and sampling data are not significantly different from each other at *p* < 0.05.

**Table 5:** Total tannins (mg/g) in grape berry skins of Touriga Nacional and Touriga Franca grape variety during different time of sampling until maturation.

Variety/Sampling Time	Control	Kaolin 5%	Potassium Silicate (0.1%)	Potassium Silicate (0.05%)
**Touriga National**				
26/07	20.01 ± 0.31 ^b^	22.42 ± 2.11 ^a^	22.04 ± 1.07 ^a^	21.23 ± 0.52 ^a^
02/08	20.25 ± 1.46 ^b^	20.42 ± 0.80 ^a^	20.19 ± 0.26 ^a^	22.82 ± 0.23 ^a^
09/08	37.39 ± 0.18 ^a^	49.24 ± 12.42 ^a^	51.28 ± 7.25 ^b^	45.53 ± 6.00 ^b^
17/08	39.99 ± 2.26 ^a^	35.71 ± 1.18 ^a^	37.66 ± 5.12 ^ab^	35.53 ± 6.75 ^ab^
23/08	32.74 ± 1.96 ^a^	34.31 ± 10.18 ^a^	35.92 ± 6.80 ^ab^	39.63 ± 2.39 ^ab^
07/09	37.31 ± 5.34 ^a^	37.80 ± 6.52 ^a^	29.93 ± 12.06 ^a^	29.26 ± 3.05 ^a^
20/09	32.36 ± 3.28 ^abA^	31.69 ± 0.57 ^abA^	31.92 ± 5.33 ^abA^	33.62 ± 2.66 ^abA^
**Touriga Franca**				
26/07	26.93 ± 0.68 ^a^	32.46 ± 1.60 ^a^	28.45 ± 2.27 ^a^	25.76 ± 1.59 ^b^
02/08	26.30 ± 2.66 ^a^	27.94 ± 1.26 ^a^	23.48 ± 0.92 ^a^	22.06 ± 0.68 ^b^
09/08	45.04 ± 19.16 ^a^	48.29 ± 7.05 ^a^	54.32 ± 11.42 ^a^	53.73 ± 3.89 ^a^
17/08	48.95 ± 0.84 ^a^	45.77 ± 1.03 ^a^	41.77 ± 6.22 ^a^	51.29 ± 3.11 ^ab^
23/08	45.78 ± 0.15 ^a^	44.12 ± 5.60 ^a^	40.22 ± 2.38 ^a^	50.43 ± 2.13 ^a^
07/09	48.68 ± 7.31 ^a^	46.37 ± 5.54 ^a^	34.79 ± 21.22 ^a^	53.27 ± 3.78 ^a^
20/09	47.59 ± 2.45 ^aA^	49.77 ±3.89 ^aA^	45.08 ± 5.42 ^aA^	39.89 ± 6.02 ^abA^

Means (average ± standard deviation) within a column followed by different lower case letters are significantly different (*p* < 0.05). Values with the same capital letter on the same line for each grape variety at final maturation data are not significantly different from each other at *p* < 0.05.

**Table 6:** Total tannins (mg/g) in grape seeds of the Touriga Nacional and the Touriga Franca grape variety during different time of sampling until maturation.

Variety/Sampling Time	Control	Kaolin 5%	Potassium Silicate (0.1%)	Potassium Silicate (0.05%)
**Touriga National**				
26/07	128.15 ± 4.29 ^b^	109.37 ± 7.38 ^a^	128.39 ± 1.23 ^a^	130.79 ± 4.46 ^a^
02/08	116.67 ± 7.95 ^b^	118.96 ± 10.24 ^a^	119.43 ± 8.17 ^a^	114.92 ± 0.54 ^a^
09/08	114.14 ± 9.08 ^a^	103.58 ± 6.77 ^a^	116.67 ± 18.68 ^b^	113.42 ± 10.66 ^b^
17/08	105.97 ± 2.29 ^a^	100.51 ± 10.45 ^a^	107.17 ± 3.02 ^ab^	104.47 ± 5.58 ^ab^
23/08	102.51 ± 0.82 ^a^	93.94 ± 6.14 ^a^	103.29 ± 4.82 ^ab^	107.62 ± 8.45 ^ab^
07/09	93.26 ± 7.22 ^a^	72.11 ± 8.11 ^a^	82.81 ± 6.16 ^a^	73.70 ± 3.99 ^a^
20/09	84.56 ± 3.62 ^abA^	78.72 ± 4.68^A^	78.15 ± 9.60 ^abA^	87.94 ± 2.66 ^abA^
**Touriga Franca**				
26/07	156.04 ± 10.58 ^a^	159.44 ± 10.19 ^a^	162.03 ± 2.63 ^a^	148.40 ± 6.49 ^b^
02/08	157.32 ± 3.52 ^a^	166.19 ± 11.83 ^a^	151.73 ± 32.69 ^a^	167.52 ± 3.47 ^b^
09/08	150.52 ± 22.83 ^a^	152.43 ± 2.00 ^a^	150.99 ± 5.36 ^a^	157.39 ± 14.89 ^a^
17/08	145.25 ± 5.13 ^a^	147.23 ± 3.97 ^a^	146.66 ± 11.66 ^a^	145.09 ± 4.69 ^ab^
23/08	129.51 ± 12.27 ^a^	134.64 ± 19.38 ^a^	126.27 ± 12.69 ^a^	129.60 ± 1.91 ^a^
07/09	105.58 ± 5.53 ^a^	128.68 ± 4.68 ^a^	114.30 ± 3.03 ^a^	121.96 ± 18.79 ^a^
20/09	104.84 ± 15.67 ^aA^	109.14 ± 4.48 ^aA^	108.37 ± 8.39 ^aA^	127.68 ± 7.28 ^abA^

Means (average ± standard deviation) within a column followed by different lower case letters are significantly different (*p* < 0.05). Values with the same capital letter on the same line for each grape variety at final maturation data are not significantly different from each other at *p* < 0.05.

**Table 7:** Total anthocyanins (mg/g) in grape berry skins of Touriga Nacional and Touriga Franca grape variety during different time of sampling until maturation.

Variety/Sampling Time	Control	Kaolin 5%	Potassium Silicate (0.1%)	Potassium Silicate (0.05%)
**Touriga National**				
26/07	0.07 ± 0.05 ^a^	0.05 ± 0.01 ^a^	0.04 ± 0.00 ^a^	0.05 ± 0.02 ^a^
02/08	0.85 ± 0.35 ^a^	0.26 ± 0.16 ^a^	0.26 ± 0.02 ^a^	0.53 ± 0.57 ^a^
09/08	3.21 ± 0.26 ^ab^	2.18 ± 0.39 ^a^	1.92 ± 0.01 ^ab^	1.89 ± 0.57 ^a^
17/08	5.39 ± 0.60 ^ab^	7.02 ± 0.65 ^b^	7.03 ± 0.13 ^bc^	7.22 ± 2.94 ^ab^
23/08	6.82 ± 2.56 ^bc^	7.63 ± 1.66 ^bc^	8.93 ± 0.44 ^c^	10.01 ± 1.41 ^b^
07/09	7.82 ± 2.71 ^c^	7.74 ± 1.56 ^c^	8.32 ± 0.11 ^c^	7.81 ± 1.44 ^b^
20/09	8.10 ± 1.49 ^cA^	8.80 ± 0.35 ^cA^	9.25 ± 0.39 ^cA^	9.42 ± 0.85 ^bA^
**Touriga Franca**				
26/07	1.55 ± 0.84 ^a^	1.13 ± 0.28 ^a^	1.43 ± 0.38 ^a^	1.67 ± 0.78 ^a^
02/08	4.75 ± 4.32 ^ab^	6.17 ± 0.51 ^ab^	5.06 ± 2.46 ^ab^	4.26 ± 3.05 ^ab^
09/08	15.02 ± 0.21 ^bc^	11.07 ± 2.93 ^abc^	9.93 ± 2.72 ^bc^	9.26 ± 5.47 ^ab^
17/08	12.33 ± 3.49 ^bc^	14.61 ± 0.66 ^bcd^	13.33 ± 1.45 ^cd^	12.63 ± 1.73 ^bc^
23/08	14.05 ± 1.85 ^c^	14.62 ± 4.99 ^bcd^	13.89 ± 3.73 ^d^	12.72 ± 0.23 ^c^
07/09	15.40 ± 2.32 ^c^	13.83 ± 1.19 ^cd^	10.27 ± 0.25 ^cd^	10.37 ± 1.91 ^bc^
20/09	10.92 ± 0.51 ^cA^	11.37 ± 0.39 ^dA^	8.59 ± 0.94 ^cdA^	8.98 ± 1.89 ^bcA^

Means (average ± standard deviation) within a column followed by different lower case letters are significantly different (*p* < 0.05). Values with the same capital letter on the same line for each grape variety at final maturation data are not significantly different from each other at *p* < 0.05.

**Table 8:** Monomeric anthocyanins (µg/g) in grape berry skins of Touriga Nacional and Touriga Franca grape varieties during different time of sampling until maturation.

Variety/Sampling Time	Control	Kaolin 5%	Potassium Silicate (0.1%)	Potassium Silicate (0.05%)
**Touriga National**				
dl-3-gluc	50 ± 0.00 ^a^	50 ± 0.00 ^ab^	50 ± 0.02 ^a^	70 ± 0.00 ^a^
cy-3-gluc	10 ± 0.00 ^a^	10 ± 0.00 ^a^	20 ± 0.00 ^a^	20 ± 0.00 ^a^
pt-3-gluc	80 ± 0.01 ^ab^	80 ± 0.00 ^ab^	80 ± 0.02 ^a^	110 ± 0.00 ^a^
pe-3-gluc	120 ± 0.02 ^ab^	140 ± 0.04 ^ab^	150 ± 0.06 ^ab^	180 ± 0.02 ^b^
mv-3-gluc	780 ± 0.05 ^abc^	780 ± 0.00 ^abc^	850 ± 0.02 ^c^	820 ± 0.00 ^bc^
cy-3-acetylgluc	20 ± 0.00 ^a^	10 ± 0.00 ^a^	20 ± 0.00 ^a^	20 ± 0.00 ^a^
pt-3-acetylgluc	10 ± 0.00 ^a^	30 ± 0.03 ^a^	30 ± 0.03 ^a^	40 ± 0.04 ^a^
pe-3-acetylgluc	30 ± 0.01 ^a^	30 ± 0.00 ^a^	40 ± 0.01 ^a^	40 ± 0.00 ^a^
mv-3-acetylgluc	250 ± 0.01 ^abc^	260 ± 0.01 ^bc^	270 ± 0.04 ^c^	230 ± 0.02 ^abc^
dl-3-coumaroylgluc	n.d.	10 ± 0.00 ^ab^	n.d.	n.d.
cy-3-coumaroyllgluc	20 ± 0.00 ^a^	20 ± 0.00 ^a^	20 ± 0.00 ^a^	20 ± 0.00 ^a^
pt-3-coumaroylgluc	20 ± 0,00 ^b^	10 ± 0.00 ^ab^	10 ± 0.01 ^ab^	n.d.
pe-3-coumaroylgluc	60 ± 0.02 ^a^	60 ± 0.01 ^a^	60 ± 0.00 ^a^	60 ± 0.00 ^a^
mv-3-coumaroylgluc	271 ± 0.01 ^a^	312 ± 0.03 ^a^	280 ± 0.05 ^a^	242 ± 0.01 ^a^
**Touriga Franca**				
dl-3-gluc	20 ± 0.00 ^b^	50 ± 0.01 ^a^	50 ± 0.01 ^ab^	60 ± 0.00 ^a^
cy-3-gluc	n.d.	10 ± 0.00 ^a^	20 ± 0.00 ^a^	10 ± 0.00 ^a^
pt-3-gluc	40 ± 0.00 ^b^	90 ± 0.00 ^a^	80 ± 0.02 ^ab^	10 ± 0.00 ^a^
pe-3-gluc	50 ± 0.01 ^a^	90 ± 0.02 ^ab^	100 ± 0.02 ^ab^	140 ± 0.01 ^ab^
mv-3-gluc	690 ± 0.01 ^a^	780 ± 0.00 ^abc^	730 ± 0.02 ^ab^	780 ± 0.02 ^abc^
cy-3-acetylgluc	10 ± 0.00 ^a^	10 ± 0.00 ^a^	10 ± 0.00 ^a^	20 ± 0.01 ^a^
pt-3-acetylgluc	10 ± 0.00 ^a^	20 ± 0.00 ^a^	10 ± 0.00 ^a^	10 ± 0.00 ^a^
pe-3-acetylgluc	10 ± 0.00 ^a^	20 ± 0.00 ^a^	20 ± 0.00 ^a^	30 ± 0.00 ^a^
mv-3-acetylgluc	200 ± 0.01 ^abc^	190 ± 0.00 ^ab^	190 ± 0.02 ^a^	190 ± 0.00 ^ab^
dl-3-coumaroylgluc	10 ± 0.00 ^bc^	10 ± 0.00 ^bc^	10 ± 0.00 ^c^	n.d.
cy-3-coumaroylgluc	20 ± 0.00 ^a^	20 ± 0.00 ^a^	20 ± 0.00 ^a^	20 ± 0.01 ^a^
pt-3-coumaroylgluc	10 ± 0.00 ^ab^	10 ±0.00 ^ab^	10 ± 0.00 ^ab^	10 ± 0.00 ^ab^
pe-3-coumaroylgluc	30 ± 0.01 ^a^	40 ± 0.00 ^a^	30 ± 0.00 ^a^	40 ± 0.01 ^a^
mv-3-coumaroylgluc	350 ± 0.03 ^a^	290 ± 0.02 ^a^	250 ± 0.03 ^a^	250 ± 0.00 ^a^

dl-3-gluc—delphinidin-3-*O*-glucoside; cy-3-gluc—cyanidin-3-*O*-glucoside; pt-3-gluc—petunidin-3-*O*-glucoside; pe-3-gluc—peonidin-3-*O*-glucoside; mv-3-gluc—malvidin3-*O*-glucoside; dl-3-acetylgluc—delphinidin-3-*O*-acetylglucoside; cy-3-acetylgluc—cyanidin-3-*O*-acetylglucoside; pt-3-acetylgluc—petonidin-3-*O*-acetylglucoside; pt-3-acetylgluc—peonidin-3-*O*-acetylglucoside; mv-3-acetylgluc—malvidin-3-*O*-acetylglucoside; dl-3-coumaroylgluc—delphinidin-3-*O*-coumaroylglucoside; cy-3-coumaroylgluc—cyanidin-3-*O*-coumaroylglucoside; pt-3-coumaroylgluc—petonidin-3-*O*-coumaroylglucoside; pe-3-coumaroylgluc—peonidin-3-*O*-coumaroylglucoside; mv-3-coumaroylgluc—malvidin-3-*O*-coumaroylglucoside. n.d—not detected, Means (average ± standard deviation) within a line followed by different letters are significantly different (*p* < 0.05).

**Table 9:** Calcium content of Touriga Nacional and Touriga Franca grape variety during different time of sampling until maturation.

Variety/Sampling Time	Control	Kaolin 5%	Potassium Silicate (0.1%)	Potassium Silicate (0.05%)
**Touriga National**				
26/07	111.5 ± 21.1 ^aA^	121.6 ± 26.6 ^aA^	130.0 ± 47.9 ^aA^	117.1 ± 26.3 ^aA^
02/08	45.9 ± 2.3 ^bA^	68.7 ± 27.6 ^aA^	73.2 ± 28.0 ^aA^	78.2 ± 57.3 ^aA^
09/08	107.2 ± 2.8 ^aA^	55.5 ± 51.4 ^aA^	66.5 ± 19.5 ^aA^	35.5 ± 15.9 ^aA^
17/08	84.6 ± 15.8 ^abA^	60.0 ± 0.7 ^aA^	85.3 ± 1.8 ^aA^	71.5 ± 11.7 ^aA^
23/08	82.2 ± 14.8 ^abA^	65.6 ± 5.4 ^aA^	57.7 ± 0.9 ^aA^	56.1 ± 5.0 ^aA^
07/09	75.3 ± 12.2 ^abA^	58.2 ± 12.6 ^aA^	75.0 ± 14.8 ^aA^	83.3 ± 16.7 ^aA^
20/09	65.0 ± 9.5 ^abA^	56.5 ± 1.9 ^aA^	69.0 ± 4.4 ^aA^	98.4 ± 7.4 ^aB^
**Touriga Franca**				
26/07	104.5 ± 13.8 ^aA^	92.9 ± 30.5 ^aA^	98.0 ± 2.0 ^aA^	78.5 ± 13.8 ^aA^
02/08	96.4 ± 50.8 ^aA^	53.7 ± 17.2 ^aA^	71.9 ± 20.8 ^aA^	79.4 ± 24.3 ^aA^
09/08	87.7 ± 13.2 ^aA^	78.4 ± 4.3 ^aA^	109.3 ± 40.7 ^aA^	160.8 ± 97.7 ^aA^
17/08	57.6 ± 16.6 ^aA^	76.1 ± 8.0 ^aA^	101.6 ± 13.7 ^aA^	86.5 ± 19.0 ^aA^
23/08	73.1 ± 11.3 ^aA^	74.6 ± 9.0 ^aA^	60.6 ± 1.1 ^aA^	73.1 ± 4.0 ^aA^
07/09	86.5 ± 1.0 ^aA^	84.2 ± 8.4 ^aA^	84.9 ± 9.8 ^aA^	89.8 ± 11.9 ^aA^
20/09	58.7 ± 16.2 ^aA^	68.6 ± 2.7 ^aA^	71.5 ± 6.7 ^aA^	67.8 ± 2.6 ^aA^

Means (average ± standard deviation) within a column followed by different lower case letters are significantly different (*p* < 0.05). Values with the same capital letter on the same line for each grape variety and sampling data are not significantly different from each other at *p* < 0.05.

**Table 10:** Potassium content of Touriga Nacional and Touriga Franca grape variety during different time of sampling until maturation.

Variety/Sampling Time	Control	Kaolin 5%	Potassium Silicate (0.1%)	Potassium Silicate (0.05%)
**Touriga National**				
26/07	646.2 ± 6.2 ^aAB^	685.0 ± 47.5 ^bcdAB^	729.7 ± 12.8 ^bcB^	568.9 ± 60.6 ^abA^
02/08	358.9 ± 25.4 ^aA^	346.1 ± 23.3 ^aA^	336.7 ± 19.7 ^aA^	468.1 ± 139.2 ^abA^
09/08	584.8 ± 343.4 ^aA^	477.8 ± 48.82 ^abA^	856.1 ± 45.7 ^bA^	879.8 ± 236.1 ^bcA^
17/08	585.9 ± 141.6 ^aA^	362.0 ± 71.5 ^aA^	358.9 ± 57.1 ^aA^	244.9 ± 63.83 ^aA^
23/08	503.7 ± 40.3 ^aA^	516.0 ± 11.5 ^abcA^	410.2 ± 65.6 ^aA^	336.3 ± 81.1 ^aA^
07/09	767.9 ± 166.0 ^aA^	861.9 ± 108.1 ^dA^	808.1 ± 39.1 ^bA^	841.1 ± 65.0 ^bcA^
20/09	839.6 ± 84.6 ^aA^	792.7 ± 110.1 ^cdA^	806.5 ± 75.9 ^bA^	1113.7± 12.8 ^cA^
**Touriga Franca**				
26/07	795.8 ± 92.0 ^aA^	782.8 ± 47.4 ^abA^	740.3 ± 104.9 ^aA^	701.0 ± 168.1 ^abcA^
02/08	382.3 ± 50.2 ^aA^	287.9 ± 82.4 ^aA^	343.2 ± 103.8 ^aA^	318.5 ± 5.7 ^aA^
09/08	629.4 ± 344.7 ^aA^	356.9 ± 38.0 ^abA^	308.6 ± 144.2 ^aA^	406.4 ± 73.3 ^abA^
17/08	502.4 ± 114.1 ^aA^	393.1 ± 57.7 ^abA^	595.8 ± 340.2 ^aA^	413.0 ± 57.8 ^abA^
23/08	468.8 ± 23.1 ^aA^	391.1 ± 13.5 ^abA^	459.9 ± 96.5 ^aA^	644.3 ± 291.8 ^abcA^
07/09	548.9 ± 64.5 ^aA^	611.1 ± 197.8 ^abA^	898.3 ± 78.9 ^abA^	1184.4 ± 386.6 ^bcA^
20/09	1084.6 ± 283.9 ^aA^	853.0 ± 265.6 ^bA^	1446.1 ± 7.3 ^bA^	1384.7 ± 167.7 ^cA^

Means (average ± standard deviation) within a column followed by different lower case letters are significantly different (*p* < 0.05). Values with the same capital letter on the same line for each grape variety and sampling data are not significantly different from each other at *p* < 0.05.

**Table 11:** Magnesium content of Touriga Nacional and Touriga Franca grape variety during different time of sampling until maturation.

Variety/Sampling Time	Control	Kaolin 5%	Potassium Silicate (0.1%)	Potassium Silicate (0.05%)
**Touriga National**				
26/07	163.9 ± 69.2 ^bA^	121.6 ± 26.6 ^aA^	223.6 ± 23.2 ^aA^	185.3 ± 16.9 ^bA^
02/08	122.5 ± 47.5 ^bA^	142.0 ± 39.4 ^aA^	166.5 ± 32.6 ^aA^	172.8 ± 28.7 ^aA^
09/08	116.8 ± 25.8 ^aA^	95.7 ± 1.5 ^aA^	129.5 ± 9.7 ^aA^	105.7 ± 2.9 ^aA^
17/08	117.7 ± 4.3 ^aA^	93.2 ± 8.2 ^bA^	112.4 ± 1.2 ^aA^	105.5 ± 28.2 ^bA^
23/08	91.2 ± 25.0 ^aA^	86.6 ± 21.2 ^aA^	80.3 ± 7.7 ^aA^	80.6 ± 1.2 ^aA^
07/09	101.1 ± 5.8 ^aA^	86.9 ± 13.0 ^bA^	127.0 ± 20.0 ^bcA^	102.0 ± 18.3 ^aA^
20/09	78.9 ± 1.5 ^aA^	83.0 ± 7.9 ^aA^	84.3 ± 1.5 ^aA^	94.5 ± 13.1 ^bA^
**Touriga Franca**				
26/07	139.9 ± 21.8 ^aA^	140.3 ± 19.8 ^aA^	147.9 ± 27.9 ^aA^	138.3 ± 39.0 ^aA^
02/08	118.0 ± 44.8 ^aA^	106.0 ± 17.6 ^aA^	154.4 ± 63.2 ^bA^	153.5 ± 4.1 ^bA^
09/08	89.5 ±3.0 ^aA^	112.1 ± 2.8 ^bA^	121.7 ± 15.8 ^bA^	137.8 ± 45.5 ^cA^
17/08	101.9 ± 19.8 ^aA^	95.6 ± 10.9 ^aA^	96.4 ± 7.8 ^aA^	103.0 ± 17.2 ^aA^
23/08	102.7 ± 15.6 ^aA^	99.2 ± 12.1 ^aA^	86.9 ± 0.7 ^aA^	92.7 ± 8.9 ^aA^
07/09	106.430.4 ^aA^	84.5 ± 7.4 ^aA^	89.7 ± 7.2 ^aA^	132.5 ± 13.9 ^bA^
20/09	83.5 ± 6.7 ^aA^	84.8 ± 2.8 ^aA^	93.3 ± 1.1 ^bA^	100.5 ± 1.3 ^bcA^

Means (average ± standard deviation) within a column followed by different lower case letters are significantly different (*p* < 0.05). Values with the same capital letter on the same line for each grape variety and sampling data are not significantly different from each other at *p* < 0.05.

## Supplementary Materials

The following are available online at https://www.mdpi.com/2079-7737/9/3/58/s1, Table S1: monthly and average annual precipitation and average temperature collected between November 2015 and October 2016.weather data for used in present study.
